# Investigation of chronic infection by *Leptospira* spp. in asymptomatic sheep slaughtered in slaughterhouse

**DOI:** 10.1371/journal.pone.0217391

**Published:** 2019-05-23

**Authors:** Daniela Santos Almeida, Lucas Nogueira Paz, Daiana Santos de Oliveira, Danielle Nascimento Silva, Paula Ristow, Camila Hamond, Federico Costa, Ricardo Wagner Portela, Alessandra Estrela-Lima, Melissa Hanzen Pinna

**Affiliations:** 1 Post Graduate Program in Animal Science in the Tropics—Federal University of Bahia., Salvador,Bahia, Brazil; 2 Instituto Gonçalo Moniz, Fundação Oswaldo Cruz, Ministério da Saúde, Salvador, Bahia, Brazil; 3 Laboratory of Bacteriology and Health, Institute of Biology, Federal University of Bahia, Salvador, Bahia, Brazil; 4 Plataforma de Salud Animal, Instituto Nacional de Investigación Agropecuaria, INIA La Estanzuela, Colonia, Uruguay; 5 Instituto de Saude Coletiva, Federal University of Bahia, Salvador, Brazil; 6 Instituto de Ciências da Saúde, Federal University of Bahia, Salvador, Brazil; Bharathidasan University, INDIA

## Abstract

The study aimed to evaluate the histopathological characteristics of renal lesions in chronically infected sheep and with low titers of anti-*Leptospira* antibodies from a slaughterhouse. In the serological analysis, 24.74% (48/194) presented seroreactivity with a titer equal to or greater than 100. Among these seroreactive sheep, titers of 100 were predominant (58.33%, 28/48), with the highest titer being 1,600 (2.08%, 1/48). Serogroup Sejroe (sv. Hardjo) was the most frequent at 35.42% (17/48). Leptospiral DNA was verified in 4.12% (8/194) of the kidney samples tested, and no urine sample was positive. All the samples corresponded to the pathogenic species *L*. *interrogans*. The eight amplicons with 202-nucleotides were identical with two mismatches (presented 100% of identity) using the PCR targeting to *secY* gene. Histological sections of PCR-positive kidneys were submitted to direct detection by the anti-LipL32 immunohistochemistry (IHC) technique. The *Leptospira* spp. antigen was evident in 62.5% (5/8) of the kidneys. Positive staining was observed in the cytoplasm of tubular cells and in the form of brownish aggregates that adhered to tubular epithelial cells and projected into the lumen. Inflammatory lymphoplasmacytic infiltrate, ranging from mild to moderate, with multifocal distribution, was the predominant finding in seroreactive animals (33.33%, 16/48). The demonstration of the leptospiral antigen lining the renal tubules through IHC of naturally infected sheep confirmed by PCR characterizes renal colonization in a species with the presence of histological changes compatible with leptospirosis.

## Introduction

Leptospires live in the wild in mammalian reservoirs, which are chronically infected in the kidneys and eliminate the bacteria in the urine, polluting the environment [1]. Rodents are considered the main carriers of the agent, among which the sewage rat (*Rattus norvegicus*) stands out as the most important reservoir in the urban ecosystems of the world [2, 3]. In rural environments, other species described as reservoirs of leptospirosis include cattles, swine, horses and canines, as well as wild animals [1, 4, 5]. Transmission occurs through direct contact with the urine of infected or indirectly from animals with contaminated environments, followed by the penetration of leptospires into skin lesions or mucous membranes [6].

Several serological investigations have shown that infection of sheep by *Leptospira* spp. is frequent and is associated in most cases with serovar Hardjo [7, 8, 9, 10]. Leptospires have been detected by direct methods in the urine and kidneys of sheep, meaning this species is susceptible to the disease and is a possible chronic reservoir of infection [8, 11].

The mechanisms of resistance or susceptibility to leptospirosis in different hosts are poorly understood and have not yet been determined, but they may be related to factors of the host or etiological agent [12]. The formation of protective biofilms in renal tubules could favor the evasion of the immune system and, consequently, chronic renal colonization, which is a process of evasion and persistence of infection, since, in biofilm, the bacteria are covered by a matrix exopolymer that protects them [13]. Brihuega et al. [14] demonstrated *in vitro* biofilm formation and *in vivo* cell aggregates from *L*. *interrogans* serovar Pomona newly isolated from a natural infection in swine.

Studies have reported the predominance of low titers of anti-*Leptospira* antibodies associated with infection in sheep flocks or samples from a slaughterhouse [15, 16, 17]. However, the microscopic agglutination test (MAT) used for serological diagnosis is not considered an adequate method to identify carriers at the individual level, since infected animals may have low or undetectable titers, necessitating the use of direct diagnostic methods for detection of carriers [18]. Leptospiral DNA has been detected in urine and different samples from live animals (urine and semen) or after slaughter (organ fragments), confirming the status of sheep as carriers [8, 9, 19, 20].

Immunohistochemistry (IHC) has been used for the detection of leptospiral antigens in several tissues [11, 21] and for the investigation of biofilm formation *in vivo* by pathogenic leptospires [22]. The present study aimed to evaluate renal colonization and the histopathological characteristics of renal lesions in asymptomatic sheep with low titers of anti-*Leptospira* antibodies from a slaughterhouse.

## Materials and methods

### Animals and samples

The protocols of the research were approved by the Ethics Committee on the Use of Animals of the Veterinary Medicine Course of the Federal University of Bahia, under number 21/2013 and were performed in accordance with Brazilian regulations for the care and use of laboratory animals.

Samples were obtained from 194 sheep sent to slaughter in a slaughterhouse, under federal inspection, located in the city of Feira de Santana, Bahia. Sampling was performed during the slaughter line, in eight visits to the slaughterhouse between January 2014 and December 2015, with a mean interval of three months between collections. Although there was no information on animal health, they had no clinical signs at the time of slaughter. During the bleeding stage, blood samples were obtained to perform the serological test. In addition, samples of urine (cystocentesis) and fragments of renal parenchyma were collected for bacteriological, histological and molecular tests.

### Bacteriological isolation

One hundred microliters of urine was inoculated into each tube containing 5 mL of EMJH liquid medium (Difco, BD, Franklin Lakes, NJ, USA), EMJH plus 300 mg/L of 5-fluoracil (5FU) [23] and Fletcher semisolid medium (Difco, BD, Franklin Lakes, NJ, USA). All preparations were supplemented with *Leptospira* Enrichment EMJH (100 ml / liter; Difco). The cultures were kept at room temperature for a maximum of 3 hours until arrival at the laboratory, where they were incubated in a BOD oven at 28°C. The cultures were examined under darkfield microscopy after 24 h and then weekly for at least 16 weeks [24].

### Microscopic agglutination test (MAT)

Blood samples from 194 animals were obtained for the detection of anti-*Leptospira* antibodies using the microscopic agglutination test (MAT), according to the World Organization for Animal Health [25] technical recommendations. A battery of live antigens from the Bacteriosis Laboratory of the Federal University of Bahia (UFBA), composed of 24 distinct serovars of leptospires, representing the 24 known serogroups [26] was used. Agglutinations were examined using darkfield microscopy (200x magnification). Titers were determined as the highest serum dilution in which at least 50% of the agglutinated leptospires were obtained for each serogroup used. Animals were considered positive when they showed titers ≥100 [25].

### Molecular identification of leptospires

DNA was extracted from the urine and kidney samples using the Wizard SV Genomic DNA Purification System (Promega, Madison, USA). In the PCR assay for the detection of the *lipL32* gene (present only in pathogenic leptospires), the primers lipL32-45F (5'-AAG CAT TAC CGC TTG TGG TG-3') and lipL32-286R (5'-GAA CTC CCA TTT CAG CGA TT-3') were used [27]. A complete protocol was recently published by Hamond et al. [28].

The *secY* housekeeping gene was amplified with the primers secYF (5′-ATGCCGATCATTTTTGCTTC-3′) and secYR (5′-CCGTCCCTTAATTTTAGACTTCTTC-3′), and nested primers secYIVF (5’-GCGATTCAGTTTAATCCTGC-3’) and SecYIV (5’-CTTAGATTTGAGCTCTAACTC-3’) with a target of 202 base pairs [29]. After amplification, the PCR products were purified and the amplicons were sequenced in both directions using the kit Big Dye Terminator 3.1 cycle sequencing (Applied Biosystems, USA) with the sequencer ABI 3500 Genetic Analyzer XL.

### Histological processing and immunohistochemistry (IHC)

Fragments of renal parenchyma measuring 2.0 x 2.0 x 0.5 cm were collected from all animals in flasks containing neutral 10% buffered formalin for fixation. Subsequently, they were processed according to the routine paraffin inclusion technique [30]. Sequential sections of 2 μm thickness were performed, which were processed for the routine staining by hematoxylin-eosin (HE). In addition, PCR-positive kidneys were also processed for anti-*Leptospira* IHC using anti-LipL32 antibody. The protocol for IHC by Croda et al. [31] was followed, with the following modification: the primary anti-LipL32 antibody and rabbit negative-control serum (for the validation of the IHC technique) were both diluted to 1:1000 in 1% BSA. For the positive control, we used a histological section of naturally infected *Rattus norvegicus* kidney from the Institute Gonçalo Moniz, Fiocruz, Bahia. Optical microscopy images were acquired using a Spot Insight Color digital camera attached to an Olympus BX-40 Microscope, using SPOT version 3.4.5 capture software and Corel DRAW software version 7.468.

For biofilm investigation, positive renal tubules in anti-LipL32 IHC were processed to co-locate the same positive tubules to the periodic acid Schiff (PAS) and Alcian blue (AA) techniques, both according to the recommendations of the commercial kit manufacturers [32, 33].

### Statistical analysis

The association between seroreactivity in MAT and the presence of renal injury was measured using the chi-square test or Fisher's exact test. To determine the risk of occurrence of histopathological changes between seroreactive and non-reactive animals in MAT, odds ratios (ORs) and their 95% confidence intervals (CIs) were calculated. The results were analyzed using Epi info 7 TM software (version 7.2.1.0, CDC Atlanta, USA).

## Results

Of the 194 sera evaluated by MAT, 24.74% (48/194) presented seroreactivity according to the cutoff titer of 100. The titers of 100 were predominant, representing 58.33% (28/48) of the positive reactions, followed by titers of 200 (33.33%, 16/48), 400 (6.25%, 3/48) and 1,600 (2.1%, 1/48). Sejroe (sv Hardjo) was the most prevalent, representing 35.42% (17/48) of the reactive samples, followed by Australis (sv Bratislava) and Pomona, both at 10.42% (5/48) (Table 1).

**Table 1:** Distribution of infective serovars and maximum titres identified by MAT in serum samples from 194 ovine animals slaughtered at slaughterhouse.

Serogroup	Serovar	Titres				Serovar specific total number (%)
		100	200	400	>800	
Sejroe	Hardjo	7	8	1	1	**17 (35.41)**
Australis	Bratislava	2	2	1	-	**5 (10.42)**
Pomona	Pomona	5	-	-	-	**5 (10.42)**
Hebdomadis	Hebdomadis	2	2	-	-	**4 (8.33)**
Ballum	Ballum	1	2	-	-	**3 (6.25)**
Icterohaemorrhagiae	Cop M20	2	1	-	-	**3 (6.25)**
Bataviae	Bataviae	2	-	-	-	**2 (4.17)**
Cynopteri	Cynopteri	2	-	-	-	**2 (4.17)**
Djasiman	Djasiman	2	-	-	-	**2 (4.17)**
Sejroe	Wolffi	-	1	1	-	**2 (4.17)**
Australis	Australis	1	-	-	-	**1 (2.08)**
Lousiana	Lousiana	1	-	-	-	**1 (2.08)**
Pyrogenes	Pyrogenes	1	-	-	-	**1 (2.08)**
**Total number of positive samples**	**n** **(%)**	**28** **(58.33)**	**16** **(33.33)**	**3** **(6.25)**	**1** **(2.08)**	**48** **(100)**

In the molecular analysis, leptospiral DNA was identified in 4.12% (8/194) of the kidney samples tested. All the samples corresponded to the pathogenic species *L*. *interrogans*. The eight amplicons were identical (presented 100% of identity) using the PCR targeting to *secY* gene. The sequences were submitted in GenBank (S1 Table). No urine samples were positive. Bacterial isolation was not obtained in the 194 urine and kidney cultures.

**Fig 1:**
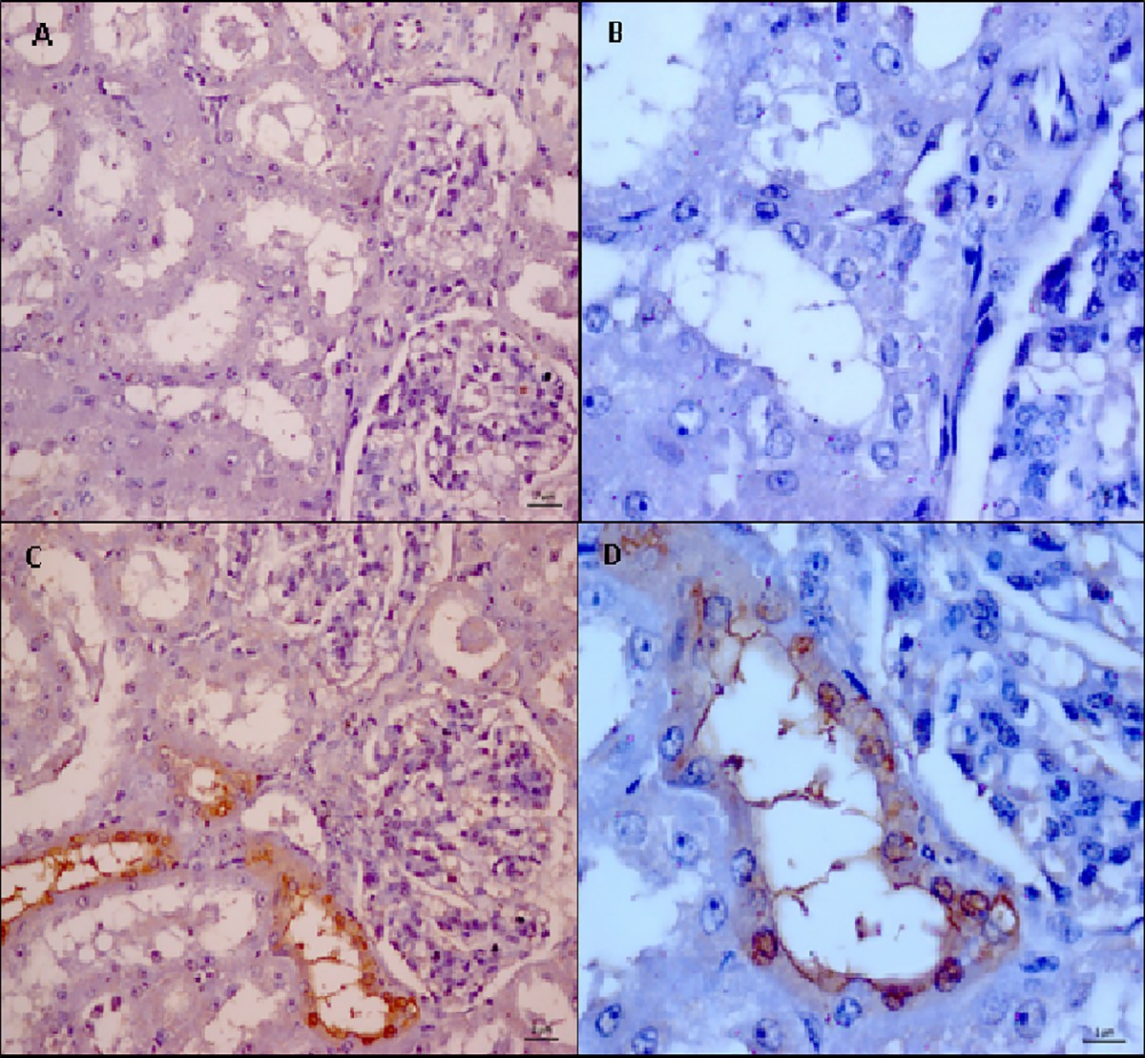
Photomicrography of renal parenchyma of sheep. (**A)** and **(B)** negative control of the reaction. Note absence of immunostaining in the epithelium and tubular lumen (magnification of 400x and 1000x respectively); (**C)** and (**D)**. Positively labeled sections by anti-LipL32: (**C)** Immunohistochemistry (IHC). Positive immunoprecipitation of *Leptospira* antigen on the epithelium and tubular lumen (magnification of 400x); (**D)** Immunostaining in tubular epithelial cells and brown-marked structures corresponding to the *Leptospira* antigen adhered to the tubular lumen (1000x magnification).

**Fig 2:**
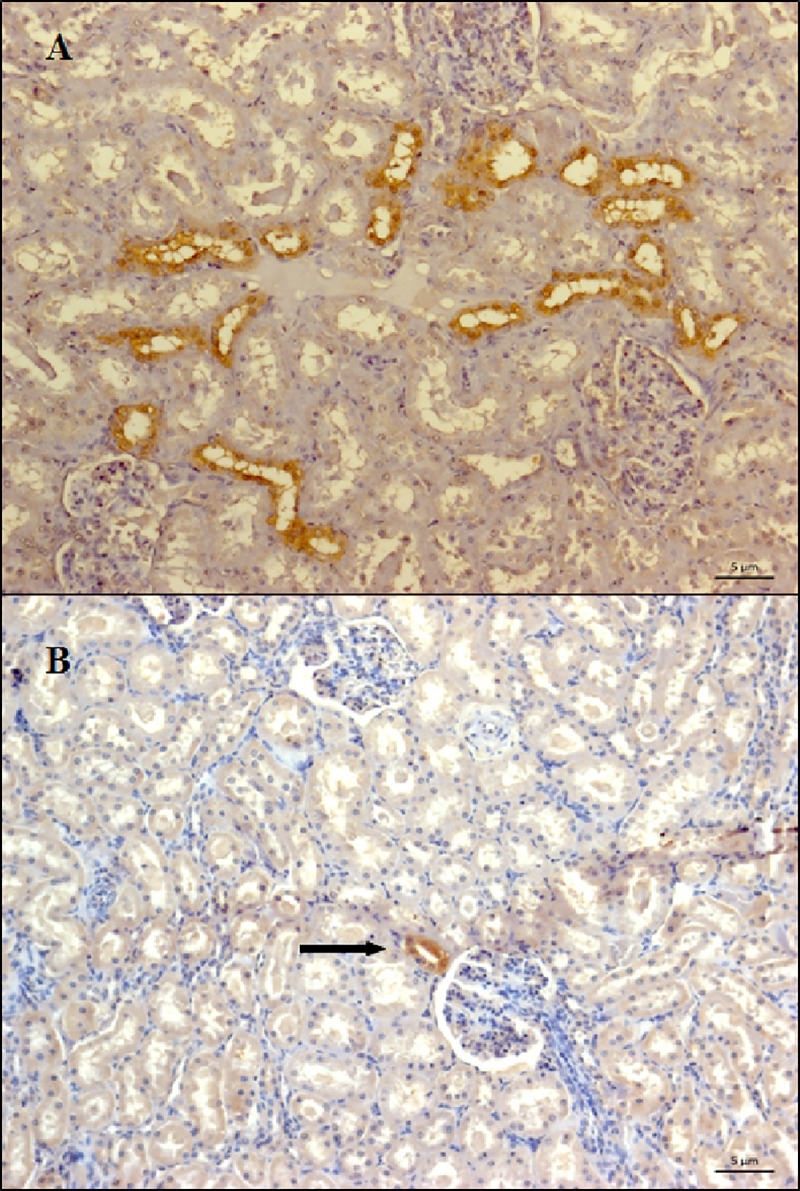
Photomicrographs of renal parenchyma of sheep with anti-protein LipL32 immunolabeling. **(A)** Mass colonization (many renal tubules marked by field) distributed throughout the cortex. (**B)** Isolated tubule labeling in the cortex (arrow) (200x magnification). In both, the *Leptospira* antigen was labeled in brown in the distal tubular epithelial cells, with non obstructive pattern.

The *Leptospira* LipL32 antigen was detected by IHC in 62.5% (5/8) of the kidney samples positive for *Leptospira* by PCR. Immunolabeling was observed in the cytoplasm of tubular epithelial cells in the form of brownish deposits that adhered to tubular epithelial cells and projected into the lumen but with a nonobstructive pattern (Fig 1). As to the distribution of the IHC-stained renal tubules, one of the animals had generalized renal colonization with marked tubules throughout the cortex, and four had one or more field-marked tubules in clusters and in different regions of the cortex (Fig 2). Alcian Blue and PAS were negative for exopolymer matrix components.

**Fig 3:**
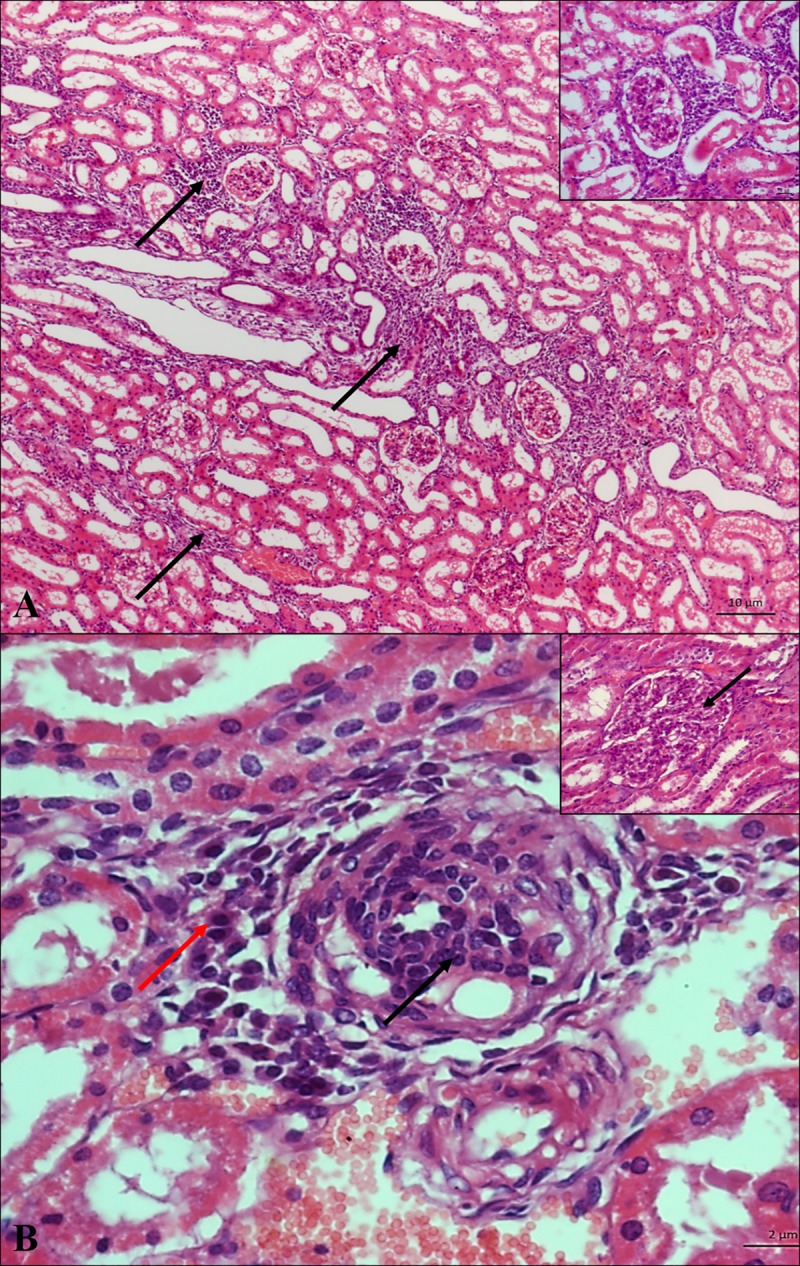
Photomicrography of renal parenchyma of HE sheep. **(A)** Interstitial nephritis (moderate) in corticomedullary region (arrows); HE (magnification 50x). **Detail:** Interstitial inflammatory mononuclear interstitial HE (magnification of 400x). (**B)** Lymphoplasmocytic infiltrate (red arrow—plasmocyte, black arrow—lymphocyte). **Detail:** Proliferative glomerulonephritis (black arrow); HE (magnification of 400x).

Macroscopically alterations were evident in only two kidneys evaluated: one presented on the surface whitish multifocal areas, with a diameter between 1 and 3 mm, that deepened in the parenchyma when cutting and another kidney was intensely congested. The histopathological analysis of the renal parenchyma of the 194 animals showed that all seroreactive (100%, 48/48) presented some type of alteration. Microscopy revealed a multifocal lymphoplasmacytic inflammatory infiltrate, ranging from mild to moderate, with a predominance of lymphocytes and a higher concentration in the cortical-medullary region (Fig 3) as the main finding in seroreactive animals (33.33%, 16/48). In addition, multifocal hydropic degeneration (10.41%, 5/48) and focal tubular necrosis (4.16%, (2/48) were found less frequently (Table 2). Non-reactive MAT and PCR negative animals, and absence of renal changes such as inflammation. Statistical analysis showed a positive association between hydropic degeneration (OR 4.12) and inflammation, represented by lymphoplasmacytic infiltrate (OR 3.21) and fibrosis (OR 3.19) in kidneys of seroreactive animals (Table 2). It should be noted that the most common finding in the kidneys of the animals that had the infection confirmed by PCR and IHC was the multifocal lymphoplasmacytic inflammatory infiltrate (Table 3).

**Table 2:** Frequency of histopathological changes and association between seroreactivity in MAT and presence of changes in seroreactive animals for *Leptospira* sp.

Type of renal injury	% of seroreactive (n = 48)	% of non-reactive (n = 146)	OR (IC)
Inflammation	33.33 (16/48)	15.06 (22/146)	**3.21 (1.52–6.77)**
Hydropic degeneration	10.41 (5/48)	4.10 (6/146)	**4.12 (1.31–12.95)**
Tubular necrosis	4.16 (2/48)	0 (0/146)	UD
Tubular ectasia	0 (0/48)	0.68 (1/146)	UD
Congestion	18.75 (9/48)	12.32 (18/146)	1.75 (0.72–4.20)
Hemorrhage	2.08 (1/48)	2.73 (4/146)	0.78 (0.08–7.16)
Regeneration	4.16 (2/48)	0 (0/146)	UD
Fibrosis	2.08% (1/48)	0.68% (1/146)	**3.19 (0.19–52.02)**
Hyaline cylinder	6.25% (3/48)	0% (0/146)	UD

UD = undefined

OR = Odds ratio

IC = Confidence interval

**Table 3:** Description of MAT results, IHC and histopathological changes in sheep kidneys positive in PCR for pathogenic leptospires.

Animal	MAT	IHC	Histopathological changes
RO 63	Bratilslava (100)	Positive	Discrete multifocal lymphocytic inflammatory infiltrate, multifocal hydropic degeneration and mild multifocal congestion
RO 70	Hardjo (100)	Positive	Lymphocytic inflammatory infiltrate and discrete multifocal congestion
RO 78	Pomona (100)	Positive	Inflammatory, multifocal, discrete infiltrate, discrete multifocal hydropic degeneration and discrete multifocal congestion
RO 161	Negative	Positive	Moderate lymphocytic inflammatory infiltrate, focal proliferative glomerulonephritis and mild focal perivasculitis
RO 162	Negative	Positive	Pronounced inflammatory infiltrate varying from multifocal to coalescing, mild multifocal tubular ectasia and mild multifocal congestion
RO 164	Negative	Negative	Focal, extensive lymphocytic inflammatory infiltrate, mild and discrete multifocal hydropic degeneration
RO 165	Negative	Negative	Discrete multifocal lymphocytic inflammatory infiltrate and discrete multifocal hydropic degeneration
RO 171	Negative	Negative	Lymphocytic inflammatory infiltrate, discrete multifocal, mild multifocal hydropic degeneration, focal necrosis and discrete multifocal congestion

## Discussion

The present study detected ovine chronic renal carriers of *L*. *interrogans* through serological, molecular and histological techniques. In this study, ovine carriers presented renal changes compatible with chronic infection characterized by the presence of lymphoplasmacytic inflammatory infiltrate. Additionally, this study is the first to demonstrate *Leptospira* immunolabeling by IHC, using anti-Lipl32 antibody in sheep and could be used as a basis for future studies aimed at elucidating the mechanisms of renal colonization and pathogenesis of leptospirosis in these animals.

Titers of 100 were the most frequent in the observed reactions. The presence of low titers can be attributed to chronic infections [11], especially when determined by serovars such as Hardjo, which result in subclinical or asymptomatic disease [5]. The serogroup Sejroe (sv Hardjo) was predominant in the seroreactive samples, as has been documented in ruminants by other authors in other Brazilian states and countries [34, 35]. According to Monahan et al. [12], during persistent renal colonization, biofilm formation could favor evasion, with low signaling to the immune system resulting in low titers of antibodies.

Leptospirosis transmission requires continuous enzootic circulation of the pathogen between animal reservoirs [36]. In this study, five of the positive animals by kidney PCR did not present seroreactivity in MAT, which reiterates the benefit of PCR to detect carriers, which often do not show any clinical signs of disease and have low titers of antibodies [37]. According to Otaka et al. [18], the microscopic agglutination test (MAT) is a good tool for screening in herds, but they did not consider it as predictor of carriers since 50% of the non-reagent MAT cattle eliminate leptospires in the urine. In environments where the disease is endemic, the occurrence of asymptomatic animals is common, and these individuals are the most important from the epidemiological point of view, since they are not identified, and they act as transmitters for other animals.

It was possible to detect pathogenic leptospiral DNA in samples of asymptomatic sheep kidneys, making them carriers, a finding that corroborates those of Barbante et al. [19], who demonstrated by the same molecular technique positivity in 12% (12/100) of the naturally infected kidney and liver samples from sheep. Additionally, Director et al.,[20] detected leptospiral DNA in 38.9% (7/18) of the urine samples from sheep. LipL32 based PCR detection has been widely applied to identify carrier animals, which are considered a source of disease spread in herds [38, 39].

Urinary excretion of leptospires may vary from species to species, from animal to animal, and from infecting serovar. Carriers may exhibit a variable period of intermittent low intensity leptospiruria, which may last up to two years or more [5]. Thus, the presence of leptospiral DNA in the kidneys does not necessarily determine the excretion at that time. We emphasize that in the present study, urine samples were collected during the animal's slaughter, making it impossible to collect subsequent samples for new molecular evaluations. In the present study, immediately after obtaining urine, PBS was added to buffer the pH of the sample and thus maintains the integrity of the possible leptospires present [40, 28]. In addition, in the present study we used positive and negative controls in all reactions. The positive controls was seeded by artificial inoculation of biological samples with leptospires before PCR assay to ensure the results.

However, the time elapsed between obtaining the samples and the molecular processing ranged from three months to two years, and they were stored in a freezer at -20° C. Additionally, the cystocentesis method of collection did not prevent the presence of mucus in most of the urine tested and is due to contraction of the urethral muscles during slaughter, with the subsequent release of semen and semen plasma. This fact may have impaired the DNA extraction process from the samples [28].

Despite the failure to obtain autochthonous leptospire strains, PCR aimed at amplifying the *lipL32* gene detected DNA from pathogenic leptospires in kidney samples, which confirms the renal carrier status. There are few reports of the isolation of leptospires from sheep in Brazil [41, 42, 43, 20]. The culture of leptospires is difficult, time-consuming and costly [42]. In addition, the culture has low sensitivity, due to the inherent difficulties of the technique, including the fastidious growth of the organism in artificial means, the contamination and the intermittent elimination of the bacteria by the hosts [44, 37].

Macroscopic alterations characterized by spots or whitish focal areas in the bovine kidneys may result from leptospire infection [45]. The changes observed in the present study include the occurrence of white-spot lesions in the kidney of a seroreactive animal that was negative by PCR but had moderate inflammatory lymphocytic infiltrate. Dorjee et al. [46] associated the presence of white spots to the serological status in sheep, with a strong relationship between the serological titer and the number of white spots in the kidneys. In a similar study, superficial white spots corresponding to areas of interstitial nephritis were identified during the inspection in 24 kidneys of cattle slaughtered in a slaughterhouse, and 19 of these showed PCR positivity with amplification of the *lipL32* gene [45].

The histological changes described in the present study are similar to those found by Carvalho and collaborators [11] in sheep and by Torres-Castro et al., [47] in rodents, in relation to the evidence of interstitial nephritis. In leptospirosis, inflammatory infiltrate is a primary alteration during acute renal injury and can be caused by direct damage by leptospires to the host tissue or by the presence of leptospiral antigen, initiating a renal immune response [36].

With the progression of infection, renal changes may vary, particularly among asymptomatic maintenance hosts, compared to symptomatic incidental hosts. Although the kidney is the organ of preference for leptospires, the pathogenesis of renal alterations is still little known in animals and especially in sheep [11]. In the present study, the inflammation in the animals positive by PCR was characterized as chronic, since the inflammatory infiltrate was composed predominantly of lymphocytes, had few plasma cells and lacked fibrosis [11].

In the present study, immunostaining adhering to tubular epithelial cells was observed in several microscopic fields in the cortico-medullary region, which corroborates the findings of Carvalho and collaborators [11], who also reported the presence of leptospires in renal tubules by IHC using anti-*Leptospira* antibody (1:400) in naturally infected sheep. Additionally, Saglam et al. [21] demonstrated *Leptospira* sp. antigen in the luminal epithelium of tubular cells and in the cytoplasm of the epithelial cells of the renal pelvis of ovine fetuses. It is worth noting that the antibody used in this study was against LipL32, which is the most abundant lipoprotein in pathogenic leptospires [48].

This absence of difference between the intensities of the histopathological lesions, without segregation between positive and negative animals in the evaluations by MAT and IHQ, can be credited to the fact that the study evaluated asymptomatic naturally infected animals, and therefore in different stages of the development of the disease. In addition, these animals may still present low or negative titers due to the chronic evolution, with low signaling for the immune system.

Even if IHC specifically labels leptospires in tissues, we emphasize that there is greater difficulty in detecting the antigen by IHC in sheep kidneys than in rat kidneys. In rats, due to the small size of the organ, it is possible to make slides with histological sections that cover the whole cortical region in a cross-section, making them representative of the organ as a whole. On the other hand, in sheep, the sections correspond to a small area (1 cm^3^) of one of the poles of the organ, which may necessitate the preparation and analysis of more than one section of parenchyma, coming from different areas of the organ, to make a reliable diagnosis. Thus, despite the positive immunoblot on the IHC, no co-localization was observed in the sequential histological sections stained with AA and PAS corresponding to the same areas. Consequently, the presence of leptospiral biofilm could not be confirmed. It should be noted that most of the evaluated animals were slaughtered at six months of age, which allows us to assume that they were potentially exposed to the agent for a short period of time, resulting in less intense colonization and no biofilm identification in our samples. In the study conducted by Santos et al. [22], adult rats were three times more likely to acquire infection than young rats. However, there is no report of a similar study in sheep. In addition, the distribution of renal colonization in naturally infected rats was heterogeneous [22]. In this study, in regard to the distribution of the stained renal tubules in the IHC, one of the animals had generalized renal colonization with stained tubules throughout the cortex, and four had one or more tubules stained by field, in agglomerates and in different regions of the cortex.

## Conclusion

Sheep detected as carriers of leptospires presented renal damage compatible with chronic infection, characterized by the presence of inflammatory lymphoplasmacytic infiltrate, which varied from mild to moderate. In these animals there was a predominance of low titers of anti-*Leptospira* antibodies and specific immunostaining of the anti-LipL32 antibody in most sheep kidneys but no biofilm was detected.

## Supporting information

S1 Table GenBank accession numbers for the consensus sequences obtained for each kidney samples from sheep included in this work. (DOCX)

S1 Text List of sequence based on secY partial gene obtained from all uncultured *Leptospira* spp. in kidney of sheep included in this work. (DOCX)
