# Supplementary material for: Investigation of chronic infection by Leptospira spp. in asymptomatic sheep slaughtered in slaughterhouse
Source: PLoS One. 2019 May 23;14(5):e0217391. doi: 10.1371/journal.pone.0217391 (PMC6532964; doi:10.1371/journal.pone.0217391)
Supplement: S1 Table — (DOCX) [file pone.0217391.s001.docx]

**S1 Table:** GenBank accession numbers for the consensus sequences obtained for each kidney samples from sheep included in this work

| **Animal ID** |  | **GenBank Accession number** |
| --- | --- | --- |
| RO63 |  | MK518377 |
| RO70 |  | MK518378 |
| RO78 |  | MK518379 |
| RO161 |  | MK518380 |
| RO162 |  | MK518381 |
| RO164 |  | MK518382 |
| RO165 |  | MK518383 |
| RO171 |  | MK518384 |
