# Supplementary material for: Investigation of chronic infection by Leptospira spp. in asymptomatic sheep slaughtered in slaughterhouse
Source: PLoS One. 2019 May 23;14(5):e0217391. doi: 10.1371/journal.pone.0217391 (PMC6532964; doi:10.1371/journal.pone.0217391)
Supplement: S1 Text — (DOCX) [file pone.0217391.s002.docx]

**S1 Text. List of sequence based on *secY* partial gene obtained from all uncultured *Leptospira* spp. in kidney of sheep included in this work**

***secY IV* sequence**

**>O63 Consensos**

AGAATTGGCTGAGAATTTGAAAAAATACGGTGGGTTCATTCCAGGAATTCGTCCGGGTTCTCACACAAAAGAATACATTGAAAAAGTGTTAAATAGAATCACTCTTCCCGGAGCTATGTTTCTTGCAGGTTTGGCATTAGCACCTTATATTATTATAAAATT

100% *L. interrogans*

**>O70 Consensos**

AGAATTGGCTGAGAATTTGAAAAAATACGGTGGGTTCATTCCAGGAATTCGTCCGGGTTCTCACACAAAAGAATACATTGAAAAAGTGTTAAATAGAATCACTCTTCCCGGAGCTATGTTTCTTGCACGTTTGGCATTAGCACCTTATATTATTATAAAATT

100% *L. interrogans*

**>O78 Consensos**

AGAATTGGCTGAGAATTTGAAAAAATACGGTGGGTTCATTCCAGGAATTCGTCCGGGGGTTCTCACACAAAAGAATACATTGAAAAAGTGTTAAATAGAATCACTCTTCCCGGAGCTATGTTTCTTGCAGGTTTGGCATTAGCACCTTATATTATTATAAAATT

100% *L. interrogans*

**>O161 Consensos**

AGAATTGGCTGAGAATTTGAAAAAATACGGTGGGTTCATTCCAGGAATTCGGTCCGGGTTCTCACACAAAAGAATACATTGAAAAAGTGTTAAATAGAATCACTCTTCCCGGAGCTATGTTTCTTGCAGGTTTGGCATTAGCACCTTATATTATTATAAAATT

100% *L. interrogans*

**>O162 Consensos**

AGAATTGGCTGAGAATTTGAAAAAATACGGTGGGTTCATTCCAGGAATTCGGTCCGGGTTCTCACACAAAAGAATACATTGAAAAAGTGTTAAATAGAATCACTCTTCCCGGAGCTATGTTTCTTGCAGGTTTGGCATTAGCACCTTATATTATTATAAAATT

100% *L. interrogans*

**>O164 Consensos**

AGAATTGGCTGAGAATTTGAAAAAATACGGTGGGTTCATTCCAGGAATTCGTCCGGGTTCTCACACAAAAGAATACATTGAAAAAGTGTTAAATAGAATCACTCTTCCCGGAGCTATGTTTCTTGCAGGTTTTGGCATTAGCACCTTATATTATTATAAAATT

100% *L. interrogans*

**>O165 Consensos**

AGAATTGGCTGAGAATTTGAAAAAATACGGTGGGTTCATTCCAGGAATTCGTCCGGGTTCTCACACAAAAGAATACATTGAAAAAGTGTTAAATAGAATCACTCTTCCCGGAGCTATGTTTCTTGCAGGTTTTGGCATTAGCACCTTATATTATTATAAAATT

100% *L. interrogans*

**>O171 Consensos**

AGAATTGGCTGAGAATTTGAAAAAATACGGTGGGTTCATTCCAGGAATTCGGTCCGGGTTCTCACACAAAAGAATACATTGAAAAAGTGTTAAATAGAATCACTCTTCCCGGAGCTATGTTTCTTGCAGGTTTGGCATTAGCACCTTATATTATTATAAAATT

100% *L. interrogans*
